# Evolution of Legislation and Crimes Based on Sexual Identity or Orientation in Spain: A Retrospective Observational Study (2011–2021)

**DOI:** 10.3390/ijerph19020859

**Published:** 2022-01-13

**Authors:** Laura Ruiz-Azcona, Amada Pellico-López, Jimena B. Manjón-Rodríguez, Mar Sánchez Movellán, Purificación Ajo Bolado, José García-Vázquez, Ildefonso Hernández-Aguado, Joaquín Cayón-De las Cuevas, María Paz-Zulueta

**Affiliations:** 1 Global Health Research Group, Departamento de Enfermería, Universidad de Cantabria, Avda, Valdecilla s/n, 39008 Santander, Spain; laura.ruiz@unican.es; 2 Departamento de Enfermería, Universidad de Cantabria, Avda, Valdecilla s/n, 39008 Santander, Spain; maria.paz@unican.es; 3 Cantabria Health Service, Avda, Derechos de la Infancia 31, 39340 Suances, Spain; 4 Hospital Comarcal de Laredo, Avda, Derechos Humanos s/n, 39770 Laredo, Spain; jimena.manjon@scsalud.es; 5 Sección de Programas de Salud de la Mujer, Dirección General de Salud Pública, Consejería de Sanidad, Gobierno de Cantabria, C/Federico Vial 13, 39009 Santander, Spain; sanchez_mm@cantabria.es (M.S.M.); ajo_p@cantabria.es (P.A.B.); 6 Consejería de Salud de Asturias, C/Ciriaco Miguel Vigil 9, 33005 Oviedo, Spain; josevazkez@yahoo.es; 7 Department of Public Health, Universidad Miguel Hernández de Elche, 03550 Alicante, Spain; ihernandez@umh.es; 8 CIBER Epidemiology and Public Health (CIBERESP), 28029 Madrid, Spain; 9 Departamento de Derecho Privado, Universidad de Cantabria, Avda de los Castros s/n, 39005 Santander, Spain; joaquin.cayon@unican.es; 10 IDIVAL, GI Derecho Sanitario y Bioética, GRIDES, C/Cardenal Herrera Oria s/n, 39011 Santander, Spain

**Keywords:** public health law, civil rights, sexual health, sexual and gender minorities, hate

## Abstract

Respect for different sexual options and orientations prevents the occurrence of hate crimes against lesbian, gay, bisexual, trans and intersex (LGTBI) persons for this reason. Our aim was to review the legislation that protects the rights of LGTBI people and to quantify the victimization rates of hate crimes based on sexual identity and orientation. A retrospective observational study was conducted across all regions of Spain from 2011–2021. The laws on LGTBI rights in each region were identified. Hate crime victimization data on sexual identity and orientation were collected in annual rates per 100,000 inhabitants, annual percentage change and average change during the study period to assess the trend. The regulatory development of laws against discrimination against LGTBI individuals is heterogeneous across regions. Overall, in Spain there is an upward trend in the number of hate crime victimizations motivated by sexual identity or orientation. The effectiveness of data collection, thanks to better training and awareness of police forces regarding hate crimes and the processes of data cleansing and consolidation contributes to a greater visibility of hate crimes against LGTBI people.

## 1. Introduction

In the definition of sexual health published by the World Health Organization (WHO) in 2001, it was already established that it requires “a positive and respectful approach to sexuality and sexual relations, as well as the possibility of having pleasurable and safe sexual experiences, free of coercion, discrimination and violence” [1].

That same year, the European Parliament passed Resolution 2001/2128 on sexual and reproductive health, making a set of recommendations to the governments of the European Union (EU) Member States. This resolution emphasizes that “sexual education should be considered in a positive and holistic way that pays attention to psycho-social as well as bio-medical aspects and based on mutual respect and responsibility”. Although reproductive health policies are the responsibility of the Member States, the EU can contribute to their improvement [2]. In Spain, to adapt the regulatory framework to the European consensus, the Organic Law 2/2010, of 3 March 2010, on sexual and reproductive health and the voluntary interruption of pregnancy was passed. In its Article 9, this law contemplates the incorporation of “sexual and reproductive health education in the educational system, as part of the integral development of education on personality and values, including a comprehensive approach”. This legislation advocates the recognition and acceptance of sexual diversity and the harmonious development of sexuality in accordance with the characteristics of young people [3].

In order to fulfill the objectives set forth in this Law, in 2011, the Spanish Government, in cooperation with the regional governments of each of the Autonomous Communities (CC.AA.) in Spain, approved the Sexual and Reproductive Health Strategy, drawn up with the collaboration of scientific and professional societies and social organizations. As a general objective in the area of sex education, this Strategy aims to “to promote quality care with accessible sexual health services, contributing to improve the experience of sexuality in an integral, autonomous, diverse, egalitarian, pleasurable, responsible, healthy and respectful manner throughout life, where the sexual and reproductive rights of women and men, regardless of their sexual options and orientations and gender identities, are guaranteed” [4]. The Strategy is based on the results of the 2009 National Survey on Sexual and Reproductive Health, according to which heterosexuality continues to be the dominant norm and latent homophobia and biphobia persist in society [5]. During the development of the Strategy, a wide variability in sexual health care between regions was noted [4].

Hate crimes have been shown to cause death, injury, illness, psychological and emotional sequelae, behavioral changes, and suicide in its victims. Respect for all options and orientations also prevents the occurrence of this type of interpersonal violence against lesbian, gay, bisexual, trans and intersex (LGTBI) persons [6,7].

In 2011, the United Nations High Commissioner for Human Rights published the report on discriminatory laws and practices and acts of violence against individuals based on their sexual orientation and gender identity. Among other practices, the following recommendation is issued “enact comprehensive anti-discrimination legislation that includes discrimination on grounds of sexual orientation and gender identity” and “investigate promptly all reported killings and other serious incidents of violence perpetrated against individuals because of their actual or perceived sexual orientation or gender identity” [8].

Evidence collected by the European Union Agency for Fundamental Rights points to concerning rates of non-reported, bias-motivated violence and harassment against minorities. The guiding principles on encouraging reporting of hate crime set out actions such as “address the invisibility of hate crime and actively communicate and disseminate hate crime data” [9].

A hate crime is defined in the Spanish Penal Code as “hostility, discrimination or violence against a group, a part of a group or against a specific person because of their membership, on racist, anti-Semitic or other grounds related to ideology, religion or beliefs, family situation, membership of an ethnic group, race or nation, national origin, sex, sexual orientation or identity, gender, illness or disability”. Hate crimes are regulated by Article 510 of the Penal Code [10]. The reform of the Organic Law 1/2015, of 30 March 2015, modified this concept [11]. The new regulation differentiates between actions of incitement to hatred or violence against groups or individuals and acts of humiliation or contempt against them and the glorification or justification of crimes committed against them or their members with a discriminatory motivation. It increases the penalties and punishes those who commit such crimes through the Internet or other social media.

The relationship between LGTBI rights legislation and related hate crimes is not sufficiently covered, mainly because hate crimes are difficult to accurately count [9,12]. Nevertheless, several international studies have shown how legislation against discrimination of LGTBI people has an overall positive impact on the reduction in hate crimes although they yield contradictory results. Thus, legislation against employment non-discrimination reduces hate crime, however, partnership recognition increases them. In addition, there are other mediating variables between LGTBI rights legislation and hate crimes as these authors argue that violence against LGTBI people is an extreme manifestation of social stigmatization and cultural norms fostered by social institutions [12].

In Spain, the Penal Code punishes hate crimes based on sexual orientation or identity and since the Organic Law 2/2010 of 3 March 2010, also those related to sexual and reproductive health [11]. The governments LGTBI of the different regions of the country have since passed laws that protect the rights of people.

The principal aim of the present study was to review the autonomic legislation regulating healthcare and lesbian, gay, trans, bisexual and intersex rights and LGTBI phobia published from the Organic Law 2/2010 until 2021. The secondary aim was to quantify the national and autonomic evolution of the main indicators of hate crimes based on sexual orientation and identity between 2015 and 2020.

## 2. Materials and Methods

### 2.1. Study Location and Population

A retrospective observational study was conducted. The study context includes all regions of Spain, differentiated by its regions and during the 2011–2021 study period.

The data sources used for each region included the official website of the Autonomous Parliament. Furthermore, the autonomic laws passed regarding healthcare and lesbian, gay, trans, bisexual and intersex rights and LGTBI phobia during the study period were identified.

The statistical data on hate crimes in Spain were collected from reports of the Ministry of the Interior based on data obtained from the Statistical System of Criminality (SEC) [13]. This computation considers the facts known by national police forces, Guardia Civil, police forces of the different autonomous communities and local police forces. Data on victimization were also collected. The concept of victimization refers to the number of facts reported by persons in which they claim to be victims or victims of a criminal offense. It differs from the concept of victim, which refers to individuals. It has been possible to collect data for the period 2015–2020 at the national level and in each Autonomous Community (including the cities of Ceuta and Melilla) on hate crimes based on sexual identity or orientation in absolute numbers.

### 2.2. Variables

Regional laws passed regulating healthcare and the rights of lesbian, gay, trans, bisexual and intersex people and LGTBI phobia during the study period. For each approved law, the following were collected: the name of the law, the date of approval, the aspects of the law related to health care, consents and regulation of the rights of minors, lesbian, gay, trans, bisexual and intersex rights.

The legislation developed in the different Spanish regions that regulate the health care and rights of LGTBI persons has been classified into four large categories. The first group comprises those regions that have regulated health care and comprehensive care for transsexual persons, including consent, hormone blocking at the onset of puberty and cross-hormonal treatment at the appropriate time of puberty to encourage their body development to match that of persons of their age, and genital reconstruction. The second group comprises the regions in which regulations focus on equality and comprehensive protection against discrimination based on sexual orientation, gender expression and gender identity including the rights of lesbian, gay, bisexual, transgender, and intersex persons; and for the eradication of homophobia, biphobia and transphobia. The third group comprises the regions that have regulated both issues. The fourth group comprises those regions that have not developed any legal text to date, neither on health care nor on the rights of LGTBI persons. Hate crime victimizations based on sexual identity or orientation are expressed as a rate per 100,000 population.

### 2.3. Data Analysis

The rates per 100,000 inhabitants of hate crime victimizations were obtained from the absolute number of victimizations, differentiated by years and regions and according to the annual population in each region. For each region and for the country as a whole, the annual percentage change in these rates and their average were calculated. The annual percentage change was estimated based on the first year (2015) and for each year the difference between each year and the previous year was calculated from the rate of the previous year (Appendix A, Table A1). The average annual percentage changes were calculated by dividing the relative change between every year by the number of years [14]. Microsoft Excel version 365 Microsoft: Washington, United States) was used to analyze the rates and trends during the study period.

## 3. Results

**Figure 1:**
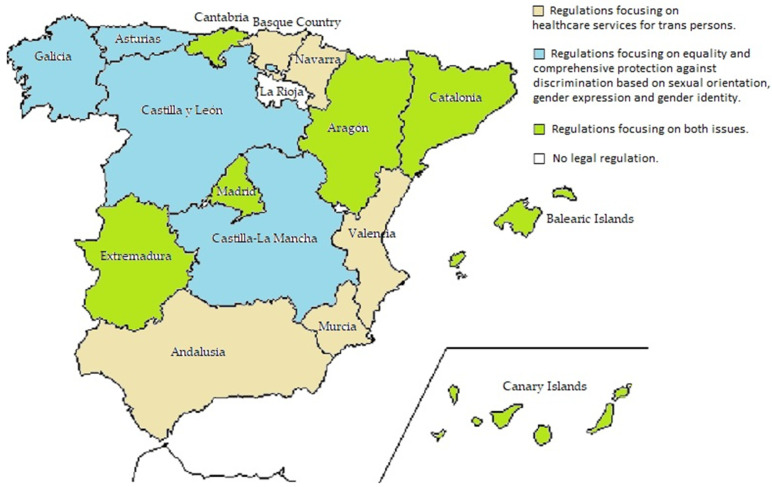
Regional laws passed regulating healthcare and the rights of LGTBI people. Spain (2011–2021).

Figure 1 shows the classification of the different regions according to the aspects legislated.

In the first group are those regions that have regulated their own healthcare services for trans persons: Andalusia, Murcia, Navarre, the Basque Country and Valencia.

In a second group are the regions in which regulations focus on equality and comprehensive protection against discrimination based on sexual orientation, gender expression and gender identity: Asturias, Galicia, Castilla-La Mancha and Castilla-León (these two are at the drafting stage).

In a third group are the regions that have regulated both issues: Aragon, Balearic Islands, Canary Islands, Cantabria, Catalonia, Extremadura and Madrid.

Finally, in La Rioja, no legal text has been developed to date, neither on health care nor on the rights of LGTBI persons.

Table 1 summarizes the results regarding the legislation developed in the different Spanish regions that regulate the health care and rights of LGTBI persons.

Considering the dates of publication and therefore the enforcement of the laws of each region, the Basque Country was the first community to legislate after the national law, publishing its regulation in 2012. The years between 2014 and 2018 were the years with the most legislation; however, even today there are regions that are still in the preliminary draft phase (Castilla-La Mancha and Castilla-León) or that lack their own regulations (Murcia).

The results represented by the number of victimizations for hate crimes based on sexual identity or orientation during the study period are shown in Table 2. The first year in which there are records of victimizations in Spain is the year 2015, when the lowest rate was recorded, whereas the highest rate was recorded in 2017; thus, an average annual percentage of change of 15.4% is observed, pointing to an upward trend.

In analyzing what occurs in the most populated regions (Andalusia, Catalonia, Madrid and Valencia), it is evident that all these regions show high rates of annual victimizations with an average annual percentage upward trend. Catalonia stands out for presenting a 49.3% average annual percentage change and the highest annual rate with 2.94 cases of victimizations per 100,000 inhabitants in 2017.

In general, an upward trend was observed in all regions, with the Balearic Islands, the autonomous city of Melilla, the Basque Country and Extremadura showing the highest annual percentage change. A downward trend was only observed in Navarra (−25.1% average annual percentage change) and Galicia (−1.2%). In La Rioja (the least populated region) and the autonomous city of Ceuta, no change was observed.

## 4. Discussion

The results regarding the development of legislation related to the rights of LGTBI individuals show that during the period from 2011 to the present, most Spanish regions have legislated both on the right to health care for trans persons and on equal treatment and non-discrimination on the basis of sexual orientation and gender identity. However, the Sexual and Reproductive Health Strategy already noted in 2011, at the beginning of the period, the wide variability between regions in sexual health care, which also seems to exist in terms of the development of legislation [4].

This variability is demonstrated by the time it has taken the different regions to develop their own regulations since the coming into force of the Organic Law 2/2010, of 3 March 2010, on sexual and reproductive health and the voluntary interruption of pregnancy, at the national level [3]. While the Basque Country legislated in 2012, other regions such as Asturias or the Canary Islands have recently published their laws, and still others are working on the draft bill. This variability also affects the content, from regions that limit themselves to regulating health care for trans people to those that provide a global approach to the protection of LGTBI rights.

Spain is among 31 of the 50 European member states of the United Nations that have criminalized incitement to hatred, violence or discrimination based on sexual orientation [33]. Compared to other member states of the European Union, it is the eight states (together with Belgium, Bulgaria, Germany, Austria, Romania, Slovenia, and Slovakia) where legislation against discrimination on grounds of sexual orientation covers not only the field of employment, but also other areas such as education, social protection, social security, and health care [34].

Levy’s study published in 2017 showed how legislation in the United States against discrimination against LGTBI people, specifically against employment discrimination, has a positive impact on reducing hate crimes against them [10]. In addition, in their study they evaluated the legislation in the different states of the country and found that this positive effect also reaches states bordering the legislating state.

Our results regarding victimization rates are negative because their number is increasing as the period progresses in Spain as a whole and in most regions, especially those with the largest populations. However, assessing the number of hate crimes reported annually and what victims reported when surveyed, we appear to be facing a serious problem of underreporting and the fact that the rates are increasing may be due to a greater visibility of the problem. In international studies, approximately one third of LGTBI people report having been victims of a hate crime in their lifetime [35,36], affecting transgender people even more [35,37]. However, victims often do not go to the police because they feel that they will not receive help [35]. In Spain, the percentage of unreported incidents has been found to exceed 89% [38].

The progression of the remaining hate crimes registered in the same period in Spain (anti-Semitism, aporophobia, religious beliefs, disability, racism, ideology and gender) shows an increase in all of them, except aporophobia, which decreased by 5.5%. In addition to crimes based on sexual identity and orientation, the most common were racism (up 10.7%), ideology (up 15.5%), disability (up 4.3%) and religious beliefs (up 2.6%) [13]. When evaluating the annual data on hate crimes for any reason in Spain, the Ministry of the Interior considers it necessary to continue to raise awareness and sensitize society, as well as the law enforcement agencies themselves, in order to continue encouraging the reporting of this type of incident and its statistical recording [39]. Hate violence can be prevented and one of the functions of public health is to analyze the magnitude of the problem through adequate case registration, epidemiological surveillance, research, and advocacy for appropriate decision making [6].

In studies based on secondary information, one of the main limitations could be a low quality of the information that could lead to a possible classification bias. To minimize these biases, we have chosen, a priori, those variables that are collected homogeneously, systematically, and objectively in the Autonomous Parliaments and the Ministry of the Interior. Data referring to hate crimes have been systematically collected and published since 2013 in Spain. From 2015 onwards, data on victimizations have been published. Year by year, progress has been made in improving the effectiveness of data collection, thanks to better training and awareness of police forces regarding hate crimes and the processes of data cleansing and consolidation [39]. Due to the limitations of the observational design of our study, it is not possible to establish causality criteria between the different variables studied (development of hate crime regulations and statistics). The conclusions of the present study are aimed at describing the development of current regional legislation on the prevention of discrimination based on sexual identity or orientation and the main indicators of hate crimes eleven years after the implementation of Organic Law 2/2010.

## 5. Conclusions

A heterogeneous development of autonomous regulations for the regulation of lesbian, gay, trans, bisexual and intersex rights and LGTBI phobia was observed. There has been a steady increase over the last six years in the number of victimization data for hate crimes based on sexual orientation or sexual identity collected by police forces. After overcoming the problem of underreporting, this increase may reflect the improvement in the effectiveness of data collection regarding hate crimes.

## Figures and Tables

**Table 1:** Autonomous Community legislation on sexual health care in each Autonomous Community during the period from 2011–2021. Chronological order.

SAC *	LGTBI ** Rights	Aspects That Are Legislated
Basque Country	2012: Law 14/2012 of 28 June [15]	Regulates the health care and rights of transgender minors.
Galicia	2014: Law 2/2014 of 14 April [16]	Regulates equal treatment and non-discrimination of lesbian, gay, transsexual, bisexual and intersex persons.
Catalonia	2014: Law 11/2014 of 10 October [17]	Regulates health care and the rights of lesbian, gay, bisexual, transgender, and intersex persons; and for the eradication of homophobia, biphobia and transphobia.
Extremadura	2015: Law 12/2015 of 8 April [18].	Regulates health care and comprehensive care for transsexual persons (including genital reconstruction) and public policies against discrimination based on sexual orientation and gender identity.
Madrid	2016: Ley 2/2016, of 29 March [19]	Regulates the protection of the right to physical, mental, sexual, and reproductive health and health care.
Murcia	2016: Law 8/2016 of 27 May [20]	Regulates the health care and rights of transsexual minors: hormone blocking at the onset of puberty and cross-hormonal treatment at the appropriate time of puberty to encourage their body development to correspond to that of persons of their age.
Balearic Islands	2016: Law 8/2016, of 30 May [21]	Regulates health care and rights of lesbian, gay, trans, bisexual and intersex persons and to eradicate LGTBI* phobia.
Valencia	2017: Law 8/2017 of 7 April [22]	Regulates the health care and rights of transsexual minors: consent, hormone blocking at the onset of puberty and cross-hormonal treatment at the appropriate time of puberty to encourage their body development to match that of persons of their age.
Navarra	2017: Foral Law 8/2017 of 19 June [23]	It regulates the health care and rights of transsexual minors: consent, hormone blocking at the onset of puberty and cross-hormonal treatment at the appropriate time of puberty to encourage their body development to match that of people their age.
Andalusia	2017: Law 8/2017 of 28 December [24]	Regulates health care and rights of transsexual minors: hormonal blockage at the onset of puberty and cross-hormonal treatment at the appropriate time of puberty to encourage their body development to correspond to that of persons of their age, in order to promote the development of desired secondary sexual characteristics.
Aragón	2018: Law 4/2018, of 19 April [25]	Regulates health care for trans persons, informed consent and shared decision making for trans minors.
	2018: Law 18/2018, of 20 December [26]	On equality and comprehensive protection against discrimination based on sexual orientation, gender expression and gender identity.
Cantabria	2020: Law 8/2020, of 11 November [27]	Regulates health care for trans and intersex persons, trans and intersex minors, social equality and non-discrimination based on sexual orientation and gender.
Asturias	2021: Decree 3/2021, of 29 January [28].	Regulates the organization and functioning of the Asturian Observatory against LGTBI * phobia.
	Preliminary draft law of the Principality of Asturias [29]	On the guarantee of the right to free expression of sexual and/or gender identity.
Canary Islands	2021: Law 2/2021 of 7 June [30]	Regulates health care for trans and intersex persons, trans and intersex minors, social equality and non-discrimination based on gender identity, gender expression and sexual characteristics.
Castilla-La Mancha	Preliminary Draft Law [31]	On sexual diversity and LGTBI rights.
Castilla y León	Preliminary Draft Bill [32]	Regulates the principle of equal treatment and non-discrimination based on sexual orientation and gender identity.
La Rioja	Not developed	

* Spanish Autonomous Communities; ** LGTBI stands for Lesbian, Gay, Bisexual, Transgender, Transsexual and Intersex.

**Table 2:** Hate crimes based on sexual identity or orientation at the national level and in each autonomous community: annual victimization rates per 100,000 inhabitants and average annual percentage of change. (Ascending order.)

	Annual Rate per 100,000 Inhabitants						
	2015	2016	2017	2018	2019	2020	Average Annual Percentage of Change
Navarra	2.50	2.50	1.24	0.31	0.00	0.60	−25.1%
Galicia	0.37	0.40	0.22	0.37	0.44	0.19	−1.2%
La Rioja	0.00	0.00	0.00	0.32	0.00	0.00	0.0%
Ceuta	0.00	0.00	0.00	0.00	0.00	0.00	0.0%
C. La Mancha	0.24	0.24	0.34	0.35	0.25	0.29	6.5%
Cantabria	0.34	0.52	0.00	0.17	0.17	0.17	10.1%
Murcia	0,27	0.14	0.34	0.34	0.20	0.26	18.0%
Valencia	0.34	0.26	0.63	0.24	0.32	0.34	18.4%
Madrid	0.30	0.65	0.60	0.64	0.60	0.62	23.2%
Castilla-León	0.28	0.16	0.29	0.21	0.67	0.33	35.5%
Catalonia	0.84	1.02	2.94	1.37	1.32	1.22	28.9%
Aragón	0.30	0.38	0.23	0.46	0.08	0.23	40.0%
Andalusia	0.17	0.39	0.21	0.49	0.50	0.41	40.6%
Asturias	0.10	0.10	0.19	0.68	0.59	0.10	51.5%
Canary Islands	0.29	0.14	0.76	0.23	0.65	0.69	99.1%
Extremadura	0.00	0.28	0.46	0.19	0.47	1.60	100.1%
Basque Country	0.87	2.42	2.14	2.41	0.54	2.75	101.6%
Melilla	0.00	3.49	1.16	2.32	1.16	8.04	115.6%
Balearic Islands	0.27	0.36	0.09	0.53	0.09	0.60	190.8%
Total Spain	0.41	0.60	0.90	0.67	0.59	0.69	15.4%

## Data Availability

Publicly available datasets were analyzed in this study. This data can be found here: http://www.interior.gob.es/web/servicios-al-ciudadano/delitos-de-odio/estadisticas (accessed on 11 September 2021).

## Appendix A

**Table A1:** Hate crimes based on sexual identity or orientation at the national level and in each autonomous community: number, population, rates per 100,000 inhabitants and average annual percentage of change (Spain, 2015–2020).

Region	Year	Number of Hate Crime Victimizations	Total Population	Rate per 100,000 Inhabitants	Annual Percentage Change (APC)	Average APC
Spain	2015	190	46,624,382	0.41	Reference	15.4%
	2016	278	46,557,008	0.60	46.5%	
	2017	419	46,572,132	0.90	50.7%	
	2018	312	46,722,980	0.67	−25.8%	
	2019	278	47,026,208	0.59	−11.5%	
	2020	328	47,450,795	0.69	16.9%	
Navarra	2015	16	640,476	2.50	Reference	−25.1%
	2016	16	640,647	2.50	0.0%	
	2017	8	643,234	1.24	−50.2%	
	2018	2	647,554	0.31	−75.2%	
	2019	0	654,214	0.00	−100.0%	
	2020	4	661,197	0.60	100.0%	
Galicia	2015	10	2,732,347	0.37	Reference	−1.2%
	2016	11	2,718,525	0.40	10.6%	
	2017	6	2,708,339	0.22	−45.2%	
	2018	10	2,701,743	0.37	67.1%	
	2019	12	2,699,499	0.44	20.1%	
	2020	5	2,701,819	0.19	−58.4%	
La Rioja	2015	0	317,053	0.00	Reference	0.0%
	2016	0	315,794	0.00	0.0%	
	2017	0	315,381	0.00	0.0%	
	2018	1	315,675	0.32	100.0%	
	2019	0	316,798	0.00	−100.0%	
	2020	0	319,914	0.00	0.0%	
Ceuta	2015	0	84,263	0.00	Reference	0.0%
	2016	0	84,519	0.00	0.0%	
	2017	0	84,959	0.00	0.0%	
	2018	0	85,144	0.00	0.0%	
	2019	0	84,777	0.00	0.0%	
	2020	0	84,202	0.00	0.0%	
C. La Mancha	2015	5	2,059,191	0.24	Reference	6.5%
	2016	5	2,041,631	0.24	0.9%	
	2017	7	2,031,479	0.34	40.7%	
	2018	7	2,026,807	0.35	0.2%	
	2019	5	2,032,863	0.25	−28.8%	
	2020	6	2,045,221	0.29	19.3%	
Cantabria	2015	2	585,179	0.34	Reference	10.1%
	2016	3	582,206	0.52	50.8%	
	2017	0	580,295	0.00	−100.0%	
	2018	1	580,229	0.17	100.0%	
	2019	1	581,078	0.17	−0.1%	
	2020	1	582,905	0.17	−0.3%	
Murcia	2015	4	1,467,288	0.27	Reference	18.0%
	2016	2	1,464,847	0.14	−49.9%	
	2017	5	1,470,273	0.34	149.1%	
	2018	5	1,478,509	0.34	−0.6%	
	2019	3	1,493.898	0.20	−40.6%	
	2020	4	1,511,251	0.26	31.8%	
Valencia	2015	17	4,980,689	0.34	Reference	18.4%
	2016	13	4,959,968	0.26	−23.2%	
	2017	31	4,941,509	0.63	139.4%	
	2018	12	4,963,703	0.24	−61.5%	
	2019	16	5,003.769	0.32	32.3%	
	2020	17	5,057,353	0.34	5.1%	
Madrid	2015	19	6,436,996	0.30	Reference	23.2%
	2016	42	6,466,996	0.65	120.0%	
	2017	39	6,507,184	0.60	−7.7%	
	2018	42	6,578,079	0.64	6.5%	
	2019	40	6,663,394	0.60	−6.0%	
	2020	42	6,779,888	0.62	3.2%	
Catalonia	2015	63	7,508,106	0.84	Reference	28.9%
	2016	77	7,522,596	1.02	22.0%	
	2017	222	7,555,830	2.94	187.0%	
	2018	104	7,600,065	1.37	−53.4%	
	2019	101	7,675,217	1.32	−3.8%	
	2020	95	7,780,479	1.22	−7.2%	
Castilla-León	2015	7	2,472,052	0.28	Reference	35.5%
	2016	4	2,447,519	0.16	−42.3%	
	2017	7	2,425,801	0.29	76.6%	
	2018	5	2,409,164	0.21	−28.1%	
	2019	16	2,399,548	0.67	221.3%	
	2020	8	2,394,918	0.33	−49.9%	
Aragón	2015	4	1,317,847	0.30	Reference	40.0%
	2016	5	1,308,563	0.38	25.9%	
	2017	3	1,308,750	0.23	−40.0%	
	2018	6	1,308,728	0.46	100.0%	
	2019	1	1,319,291	0.08	−83.5%	
	2020	3	1,329,391	0.23	197.7%	
Andalusia	2015	14	8,399.043	0.17	Reference	40.6%
	2016	33	8,388,107	0.39	136.0%	
	2017	18	8,379,820	0.21	−45.4%	
	2018	41	8,384,408	0.49	127.7%	
	2019	42	8,414,240	0.50	2.1%	
	2020	35	8,464,411	0.41	−17.2%	
Asturias	2015	1	1,051,229	0.10	Reference	51.5%
	2016	1	1,042,608	0.10	0.8%	
	2017	2	1,034,960	0.19	101.5%	
	2018	7	1,028,244	0.68	252.3%	
	2019	6	1,022,800	0.59	−13.8%	
	2020	1	1,018,784	0.10	−83.3%	
Canary Islands	2015	6	2,100,306	0.29	Reference	99.1%
	2016	3	2,101,924	0.14	−50.0%	
	2017	16	2,108,121	0.76	431.8%	
	2018	5	2,127,685	0.23	−69.0%	
	2019	14	2,153,389	0.65	176.7%	
	2020	15	2,175,952	0.69	6.0%	
Extremadura	2015	0	1,092,997	0.00	Reference	100.1%
	2016	3	1,087,778	0.28	100.0%	
	2017	5	1,079,920	0.46	67.9%	
	2018	2	1,072,863	0.19	−59.7%	
	2019	5	1,067,710	0.47	151.2%	
	2020	17	1,063,987	1.60	241.2%	
Basque Country	2015	19	2,189,257	0.87	Reference	101.6%
	2016	53	2,189,534	2.42	178.9%	
	2017	47	2,194,158	2.14	−11.5%	
	2018	53	2,199,088	2.41	12.5%	
	2019	12	2,207,776	0.54	−77.4%	
	2020	61	2,220,504	2.75	405.4%	
Melilla	2015	0	85,584	0.00	Reference	115.6%
	2016	3	86,026	3.49	0.0%	
	2017	1	86,120	1.16	−66.7%	
	2018	2	86,384	2.32	99.4%	
	2019	1	86,487	1.16	−50.1%	
	2020	7	87,076	8.04	595.3%	
Balearic Islands	2015	3	1,104,479	0.27	Reference	190.8%
	2016	4	1,107,220	0.36	33.0%	
	2017	1	1,115,999	0.09	−75.2%	
	2018	6	1,128,908	0.53	493.1%	
	2019	1	1,149,460	0.09	−83.6%	
	2020	7	1,171,543	0.60	586.8%	
